# Electrodeposition of p-Type Sb_2_Te_3_ Films and Micro-Pillar Arrays in a Multi-Channel Glass Template

**DOI:** 10.3390/ma11071194

**Published:** 2018-07-12

**Authors:** Ning Su, Shuai Guo, Fu Li, Dawei Liu, Bo Li

**Affiliations:** 1 Graduate School of Shenzhen, Tsinghua University, Shenzhen 518065, China; 18842686901@163.com (N.S.); gs15@mails.tsinghua.edu.cn (S.G.); 2 Shenzhen Key Laboratory of Advanced Thin Films and Applications, College of Physics and Energy, Shenzhen University, Shenzhen 518060, China; 3 Huaneng Clean Energy Research Institute, Beijing 102209, China; liudawei@hnceri.com

**Keywords:** thermoelectric device, electrodeposition, Sb_2_Te_3_ micro-pillar arrays, multi-channel glass template

## Abstract

Antimony telluride (Sb_2_Te_3_)-based two-dimensional films and micro-pillar arrays are fabricated by electrochemical deposition from electrolytes containing SbO^+^ and HTeO_2_^+^ on Si wafer-based Pt electrode and multi-channel glass templates, respectively. The results indicate that the addition of tartaric acid increases the solubility of SbO^+^ in acidic solution. The compositions of deposits depend on the electrolyte concentration, and the micro morphologies rely on the reduction potential. Regarding the electrolyte containing 8 mM of SbO^+^ and 12 mM of HTeO_2_^+^, the grain size increases and the density of films decreases as the deposition potential shifts from −100 mV to −400 mV. Sb_2_Te_3_ film with nominal composition and dense morphology can be obtained by using a deposition potential of −300 mV. However, this condition is not suitable for the deposition of Sb_2_Te_3_ micro-pillar arrays on the multi-channel glass templates because of its drastic concentration polarization. Nevertheless, it is found that the pulsed voltage deposition is an effective way to solve this problem. A deposition potential of −280 mV and a dissolve potential of 500 mV were selected, and the deposition of micro-pillars in a large aspect ratio and at high density can be realized. The deposition technology can be further applied in the fabrication of micro-TEGs with large output voltage and power.

## 1. Introduction

Thermoelectric (TE) devices, which can directly and reversibly convert heat into electricity, have attracted worldwide interest because of their promising applications in electronics, which arise from their distinct characteristics, such as no mechanical moving parts, no maintenance, and high reliability [1,2,3,4,5,6,7,8,9,10,11,12,13,14,15,16,17]. In particular, one of their unique advantages compared with traditional energy conversion stems is their very high specific power, which can scale well to low power levels and be useful in applications in which weight and size matter. Thus, miniaturized TE devices will display favorable and efficient performance. Technology to miniaturize and integrate TE devices has become an important research direction with which to promote their materialization. Many advances in the fabrication of microscopic TE devices combined with microelectromechanical systems processing have been achieved in recent years. However, there are still some important problems that have limited the performance and application of TE micro-devices; for instance, the complex mic-fabrication process, the quality of TE materials including thin films and microarrays, the aspect ratio of microarrays, and contact resistance [3,9,10,12,14,18,19,20,21,22,23,24,25,26]. 

Typically, TE modules consist of a series of electrically connected p- and n-type elements. In micro-devices, the basic components are the corresponding p- and n-type TE thin films or microarrays. Thus, the deposition of both films and microarrays is an important technology with which to obtain high-performance devices. Bismuth telluride (Bi_2_Te_3_)-based alloys are the best n-type TE materials near room temperature and the corresponding films and microarrays have been studied extensively in TE micro-devices. The electrodeposition of Bi_2_Te_3_ films has been well reported because electrochemical deposition has many advantages including comparably facile control over process parameters, low cost, low temperature, and relatively simple equipment requirements [4,9,14,17,22,25,27,28,29,30]. Additionally, electrodeposited materials can be integrated into micro-devices. Considering the compatibility factor of TE materials that influences the output power of the device, p-type antimony telluride (Sb_2_Te_3_) is a suitable TE material to match with n-type Bi_2_Te_3_ [5,30,31,32,33,34,35]. However, few Sb_2_Te_3_ films prepared by electrochemical deposition have been reported, mainly because SbO^+^ is difficult to dissolve in acidic solution, which leads to poor crystallization of the pure Sb_2_Te_3_ and weak TE properties of the final films. Meanwhile, few studies on p-type Sb_2_Te_3_ microarrays are available [9,29,36,37,38]. Especially, Sb_2_Te_3_ microarrays with high aspect ratio are difficult to obtain. Therefore, it is hard to obtain a large temperature difference between two ends of the legs, resulting in a lower output power of the final micro-device.

In this work, electrodeposition of Sb_2_Te_3_ thin films on silicon (Si) wafer surface and microarrays on a multi-channel glass template are investigated. Electrodeposition on Si wafer surface-based Pt electrode is firstly carried out to clarify the possibility and process of Sb_2_Te_3_ deposition, which also acts as a reference to subsequently fabricate microarrays by electrodeposition, which are used in TE devices. Actually, in the present study, the microarrays for TE micro-devices are mainly obtained by deposition in Si micro-pore, anodic aluminum oxide (AAO), and organic photoresist templates [20,39,40,41,42,43]. However, the height of the micro-pores in the AAO and organic photoresist templates is limited by the processing technique, which induces a lower aspect ratio of the micro-pillars. Thus, it is difficult to increase the temperature difference between two ends of the micro-device, leading to lower conversion output power and efficiency. Moreover, the thermal conductivity of the AAO and Si templates is higher than that of TE materials. Thus, they must be removed after deposition to guarantee the energy conversion efficiency of the TE device. However, removing the micro-pore template degrades the mechanical properties of the microarrays, which tends to break the microarrays easily without the support. Therefore, here we use multi-channel glass as the template for the Sb_2_Te_3_ electrodeposition after optimization of the deposition parameters by using the films. The glass template has low thermal conductivity and is electrically insulating. It can be retained after being filled with TE materials, which simplifies the device fabrication process and also helps to enhance the rigidity of the TE modules. Notably, the multi-channel glass template used in the present work is fabricated by laser boring. Although the template aspect ratio is about 4:1 in this work, it can easily be further increased to obtain a high aspect ratio by increasing the thickness of the initial glass substrate.

## 2. Materials and Methods

Sb-Te films were deposited from the nitric acid (3%) (Guanghua Technology Co. Ltd., Shenzhen, China)- and tartaric acid (20 g/L) (Macklin Biochemical Co. Ltd., Shanghai, China)-supporting electrolyte containing Sb^+^ and HTeO_2_^+^ in a three-electrode system with a capacity of 0.2 L at room temperature. The electrodeposition reaction of Sb_2_Te_3_ can be expressed as shown in Equation (1) [36].

2SbO^+^ + 3HTeO_2_^+^ + 13H^+^ + 18e^−^→Sb_2_Te_3_ + 8H_2_O (1)

The anode was polished graphite, which remained chemically stable during the deposition in acidic solution. The cathode was a Si wafer that was coated with 20 nm of Ti and 150 nm of Pt. The wafer was then cleaved into pieces with dimensions of 10 × 10 mm. To remove any organic materials from the Si wafer, it was pre-cleaned with a 2:1 mixture of concentrated sulfuric acid and hydrogen peroxide. The reference electrode was potassium chloride saturated calomel electrode (SCE), which had a constant potential of 0.2412 V, and the potential difference between the cathode and SCE could be observed by an electrochemical workstation (CHI-660E, CH Instruments Co. Ltd., Shanghai, China). All stated potentials are relative to SCE. The deposition process was controlled by a personal computer and the electrochemical workstation. Deposition on Si wafer-based Pt electrode was carried out firstly to determine the optimal electrolyte concentration and deposition potential. The electrolyte contained 8 mM of Sb^+^ and 8 to 15 mM of HTeO_2_^+^. Electrolyte pH was controlled at −0.1~0.1 with nitric acid according to the potential–pH graph. Deposition and dissolution peak potentials were observed by cyclic voltammetry at a scan rate of 5 mV/s. Then, Sb-Te films were fabricated by constant voltage processes and their microstructure, composition, crystal structure density, and electrical properties were investigated. The electrical resistivity and Seebeck coefficient of the films as function of temperature from 310 K to 417 K in helium atmosphere were measured by using the equipment (ZEM-3, ULVAC-RIKO, Yokohama, Japan).

The optimized electrolyte concentration and potential of the Sb_2_Te_3_ films were tried to fabricate the microarrays based on the multi-channel glass. The multi-channel glass template was fabricated from alkali-free glass. Arrays of vertical holes with a diameter of 0.06 mm and depth of 0.2 mm were formed by a laser. The distance between neighboring holes was 0.2 mm. A complete template contained about 2500 holes. The template was then attached onto a Si wafer by using polyethylene terephthalate (PET) tape for the next deposition. Morphologies and chemical composition of all the Sb-Te deposited films and arrays were investigated by scanning electron microscopy (SEM; Zeiss SUPRA^®^ 55, Berlin, Germany) and energy-dispersive spectroscopy (EDS; Oxford X-Max 20, Oxford, UK). The crystalline structure and composition of deposits were analyzed by X-ray diffraction (XRD; Rigaku, Tokyo, Japan), which is conducted using a Rint-2000V/PC diffractometer in 2θ mode with Cu-Kα radiation. Then, a microprobe test platform (RTS-9, four probes technology Co. LED, Guangzhou, China) was used to measure the potential–current curve of a single pillar to calculate its resistance.

## 3. Results

### 3.1. Electrodeposition on Si Wafer-Based Pt Electrode

**Figure 1:**
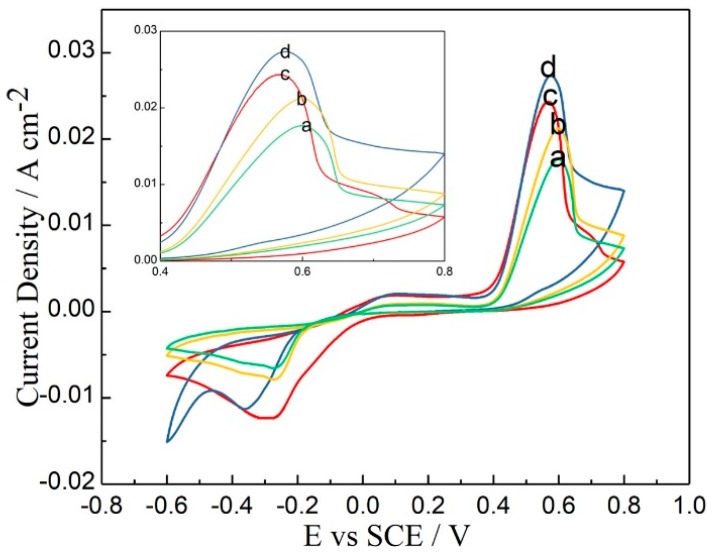
Cyclic voltammetry scans of the Sb-Te films at room temperature at a scan rate of 0.05 V/s with electrolytes containing 8 mM of SbO^+^ and (**a**) 8, (**b**) 10, (**c**) 12, and (**d**) 15 mM of HTeO_2_^+^.

Sb-Te films can be deposited on the cathode in an acidic electrolyte of SbO^+^ and HTeO_2_^+^ [29,44,45]. Some reports indicated that Sb was difficult to dissolve in acidic solution in the absence of complexing agents. Tartaric acid can promote the dissolution process of Sb and stabilize the solution system, because it chelates strongly to Sb. When Sb was added to nitric acid solution, a white precipitate formed immediately. As tartaric acid was added to the mixture, the white precipitate gradually disappeared. The dissolution rate was relative to the amount of tartaric acid added. Cyclic voltammetry scans were conducted in electrolytes containing 8 mM of SbO^+^ and 8~15 mM of HTeO_2_^+^ at a scan rate of 5 mV/s at room temperature to investigate the electrochemical behavior of the Si wafer. As shown in Figure 1, every cyclic voltammogram contains only a reduction peak and an oxidation peak, which represent the deposition and dissolution processes of Sb-Te, respectively. This behavior indicates that the crystallization and nucleation processes of Sb_2_Te_3_ on the Si wafer-based Pt electrode occurred at the same time, rather than Sb or Te nucleating first and then inducing the growth of Sb_2_Te_3_. The oxidation peak potential was approximately 0.6 V, and the reduction peak potential ranged from −0.25 to −0.35 V as the Te concentration of the electrolyte changed from 8 to 15 mM. The charge integral and current density of the deposition and dissolution processes both increased with Te concentration, which indicated that the reaction rate was related to the Te concentration. The electrodeposition reaction occurred more easily at lower over-potential, which corresponded to a more positive reduction peak potential.

**Figure 2:**
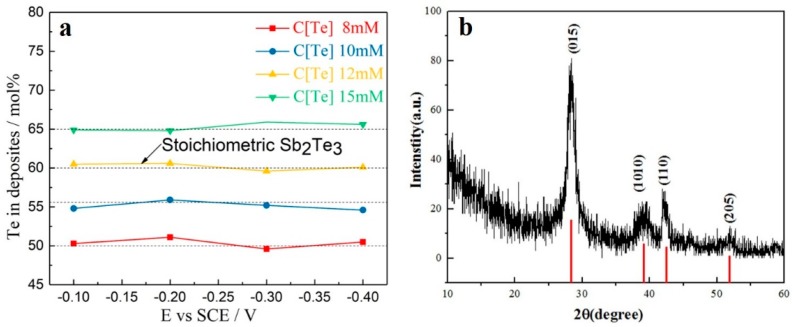
(**a**) EDS results for films deposited with electrolyte consisting of 8 mM of SbO^+^ and 8–15 mM of HTeO_2_^+^; (**b**) XRD pattern of the film deposited in 8 mM of SbO^+^ and 12 mM of HTeO_2_^+^ at a potential of −0.3 V.

The EDS and XRD results of the films deposited from electrolytes containing 8 mM of SbO^+^ and 8–15 mM of HTeO_2_^+^ were investigated. As illustrated in Figure 2a, the composition of the deposits was strongly related to the HTeO_2_^+^ concentration of the electrolyte, but had almost no relationship with deposition potential. This is probably because HTeO_2_^+^ adsorbed strongly on the Pt electrode. Therefore, the composition of the deposits near the electrode depended on the adsorption capacity of HTeO_2_^+^, rather than the over-potential during deposition. In addition, the composition of the film can be calculated by Equation (2),

X_Te_ = (i_Te_/z_Te_)/{(i_Sb_/z_Sb_) + (i_Te_/z_Te_)} (2)

In which X_Te_ is the content of Te in deposits, i_Sb_ and i_Te_ are the limited current values of the Sb(III) and Te(IV), respectively, and z_Sb_ = 3 and z_Te_ = 4 are the valences of Sb and Te, respectively. i_Sb_, i_Te_, z_Sb_, and z_Te_ are related to the concentration of electrolyte, but no relationship with deposition potential. This conclusion is consistent with some of the reports [46,47,48,49,50,51]. When deposited from the electrolyte containing 8 mM of SbO^+^ and 12 mM of HTeO_2_^+^, the i_Sb_ = −4.59 × 10^−4^ Acm^−2^ and i_Te_ = −9.2 × 10^−4^ Acm^−2^, and the X_Te_ = 60 mol % can be calculated. The electrodeposition process could be divided into two steps. The first was the adsorption of HTeO_2_^+^ on the Pt substrate, which could be expressed as HTeO_2_^+^→ (HTeO_2_^+^)_ads_. The adsorbed HTeO_2_^+^ ion on the Si wafer based Pt electrode then reacted with SbO^+^ in solution, producing the final compound of Sb_2_Te_3_. The percentage of Te in the films increased with the HTeO_2_^+^ concentration of the electrolyte. Nearly stoichiometric Sb_2_Te_3_ was obtained by electrodeposition using an electrolyte consisting of 8 mM of SbO^+^ and 12 mM of HTeO_2_^+^ scanning at a potential range from −0.1 to −0.4 V for 600 s. The XRD pattern of the film deposited in 8 mM SbO^+^ and 12 mM HTeO_2_^+^ at a potential of −0.3 V is shown in Figure 2b. This pattern confirms that the deposit is Sb_2_Te_3_ (JCPDS # 15-0874). Considering the results of electrochemical measurements and the EDS and XRD analyses, we used an electrolyte with 8 mM of SbO^+^ and 12 mM of HTeO_2_^+^ in subsequent experiments.

**Figure 3:**
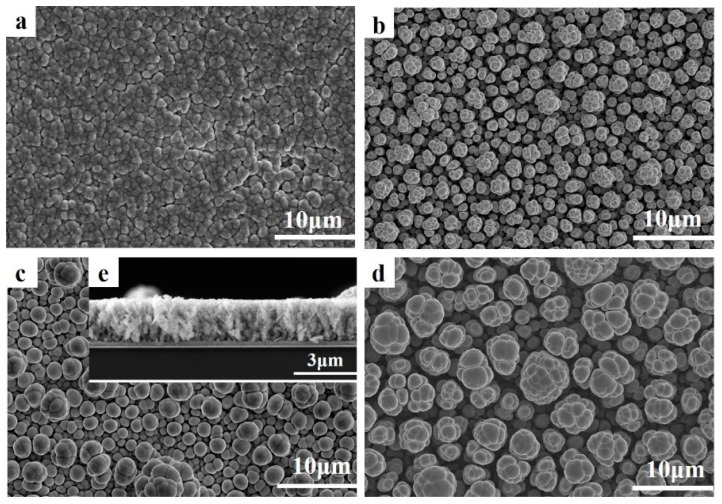
Top-view SEM images of Sb-Te films deposited on Si wafer-based Pt electrode under a constant applied voltage of (**a**) −100, (**b**) −200, (**c**) −300, and (**d**) −400 mV; (**e**) cross-sectional view of the film deposited at −300 mV.

The deposition process on a Si wafer-based Pt electrode was then observed. After about 60 s, the surface of the Pt electrode became gray and the whole surface exhibited a metallic sheen. As the deposition time increased, the films got thicker and denser [41]. The thin films deposited from the electrolyte containing 8 mM of SbO^+^ and 12 mM of HTeO_2_^+^ at potentials of −100 to −400 mV were investigated by SEM. Figure 3 shows the top views of all the films and cross-section for the film prepared at potentials of −300 mV. The top view reveals that Sb_2_Te_3_ consisted of equiaxed globular grains, with small grains uniting to form larger spheres [46], which is different from that of laminar Bi_2_Te_3_. At a potential of −100 mV, the crystal grains were smaller, and the film was much more compact than that deposited at a more negative potential. As the deposition potential becomes negative, the grains size increases, and the films become less dense owing to the larger growth rate resulting from larger over-potential. The films deposited at −100 and −400 mV were much thinner than those deposited at −200 and −300 mV in the same time of 20 min, because the deposition current at the deposition potential of −100 and −400 mV is smaller than that at −200 and −300 mV, which we could obtain from the cyclic voltammetry curves. The longitudinal section views for the film deposited at −300 mV at 20 min reveal that the globular crystals of Sb_2_Te_3_ stack along the normal direction of the Pt electrode, and the crystals close to the Pt electrode are much smaller than those close to the surface. The surface of the film is smooth, and the film thickness is about 2.5 µm.

These results suggest that the micromorphology and composition of deposits are related to both the concentration of HTeO_2_^+^ in electrolyte and reduction potential. Sb_2_Te_3_ film with nominal composition and dense morphology in the present work can be obtained using reduction potential of −300 mV with 8 mM of SbO^+^ and 12 mM of HTeO_2_^+^ in subsequent.

**Figure 4:**
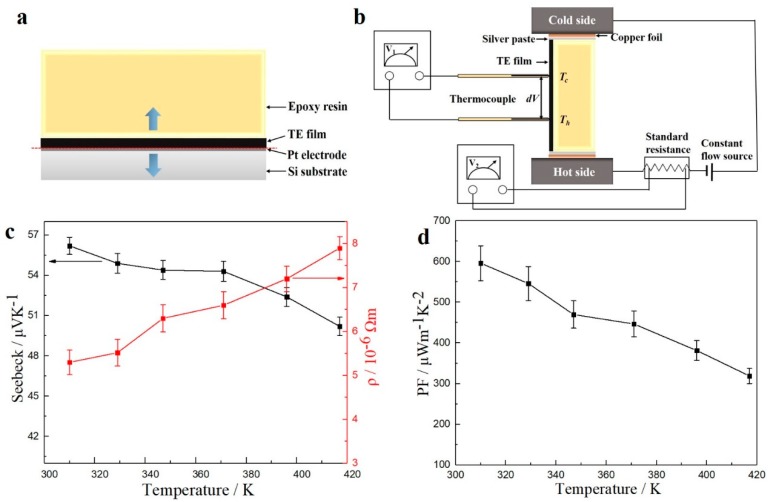
(**a**,**b**) The schematic diagram of testing method for electrical properties for Te thin films deposited at −300 mV from the electrolyte of 8 mM SbO^+^ and 12 mM HTeO_2_^+^. Electrical resistivity and Seebeck coefficient (**c**) and power factor (**d**) values of TE film as function of temperature from 310 K to 417 K in helium atmosphere.

The electrical resistivity and Seebeck coefficient of the film deposited at −300 mV from the electrolyte of 8 mM SbO^+^ and 12 mM HTeO_2_^+^ were measured using ZEM-3 equipment, because the films were in direct contact with Pt electrode, which has higher electrical and thermal conductivity and significant influence on the electrical testing results. The films must be removed to another substrate, which is electric insulation and has lower thermal conductivity. As shown in Figure 4a, epoxy resin was applied on the surface of films, and it solidified after a few hours, which allowed it to pick off the films. Then, the sample was placed between the hot and cold sides of ZEM, and the electrical connection was realized by silver paste and copper foil, which is shown in Figure 4b. Figure 4c,d shows the electrical conductivity, Seebeck coefficient, and power factor values of the aforementioned films (deposited at −300 mV from the electrolyte of 8 mM SbO^+^ and 12 mM HTeO_2_^+^) as function of temperature, respectively, because the inferior heat resistance of the epoxy resin, which was only slightly below 417 K, was tested. The Seebeck coefficient is positive, which proves that the deposit is p-type semiconductor. Seebeck coefficient decreases, and the electrical conductivity increases with the increasing temperature. Power factor value reaches its maximum of 595.93 μW/mK^2^ at 310 K. The Seebeck coefficient and electrical resistivity have strong relationship with the quality of deposits. The Seebeck coefficient of our TE films is 56.2~50.2 μV/K, and the electrical resistivity is 5.3 × 10^−6^~7.9 × 10^−6^ Ωm at a temperature of 310~417 K, which is similar to the TE films in previous study [36,47,48]. However, the Seebeck coefficient of the TE films is far below the bulk materials, on account of the fact that the TE films are much looser than bulk materials. 

### 3.2. Electrodeposition onto Multi-Channel Glass Templates

**Figure 5:**
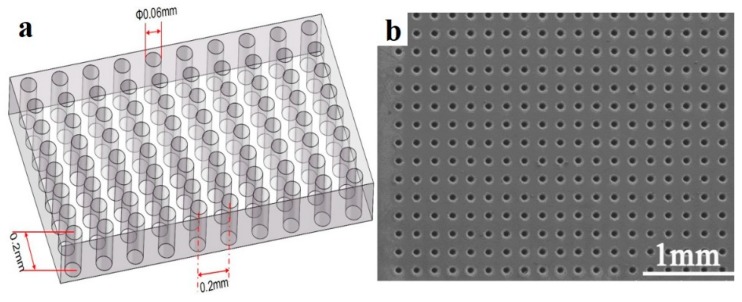
(**a**) Schematic and (**b**) SEM image of the multi-channel glass template.

**Figure 6:**
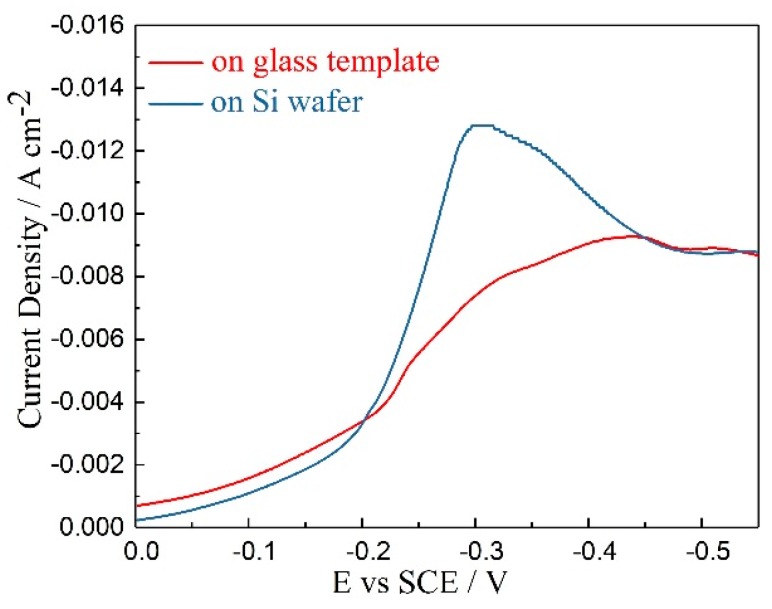
Cyclic voltammograms of Sb-Te electrodeposition on the Si wafer-based Pt electrode and glass template at room temperature at a scan rate of 5 mV/s with an electrolyte containing 8 mM of SbO^+^ and 12 mM of HTeO_2_^+^.

Figure 5 displays the schematic and scanning electron micrographs of the multi-channel glass template. During the deposition process, the template was attached to an Si wafer by PET gum, which has high chemical stability and viscosity in acidic solution. Cyclic voltammetry scans were conducted to observe the electrodeposition behavior on both an Si wafer and glass template; these scans are compared in Figure 6. The curves were obtained in an electrolyte with 8 mM of SbO^+^ and 12 mM of HTeO_2_^+^ at a scan rate of 5 mV/s at room temperature from 0 to −0.55 V. The linear voltammograms revealed that the reduction peak of the glass template was not as obvious as that for the Si wafer. The current density of the deposition process on the glass template is lower than that on the Si wafer. This indicates that the deposition process is more difficult and requires a larger driving force for the glass template than for the Si wafer. Therefore, much higher over-potential is needed to promote the reaction on the glass template compared with that on the Si wafer.

**Figure 7:**
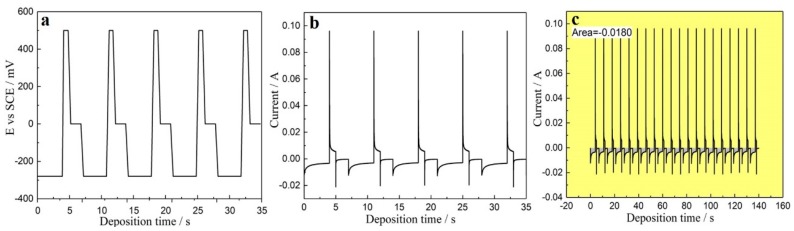
(**a**) Three-step pulsed voltage-time curve, (**b**) current-time curve, and (**c**) integral charge of deposits under pulsed voltage conditions.

By adjusting the potential, a high-quality film can be obtained on the Si wafer-based Pt electrode. In contrast, it was difficult to obtain compact pillars on the glass template. We found that the deposit density was too low to satisfy the requirements of TE modules. This is because the micro-pores on the glass template were rather deep and their large aspect ratio led to drastic concentration polarization. In addition, the ions in solution diffuse poorly, because the deposition reaction can only occur on the Pt electrode, which is located at the bottom of the micro-pores. Pulsed voltage deposition was therefore investigated to solve the problem of concentration polarization [3,36,52]. Figure 7 shows the three-step pulsed voltage-time and current-time curves for this deposition experiment. At the beginning of every deposition stage, the current density rapidly reached a very high value, then dropped abruptly and gradually tended to smooth, eventually reached a steady state. At the beginning, the current density rapidly increases, corresponding to a quickly nucleated process on the surface of the substrate. As the number of crystal nucleus increases, the diffusion cross-section projection of the solute near the substrate also increases, corresponding to the acceleration of crystal growth, that is, the increase of the current; subsequently, the current value slowly declines and finally reaches stability, corresponding to the formation of concentration polarization. Eventually, the concentration gradient of electrolyte nearing the electrode gradually becomes stable.

**Figure 8:**
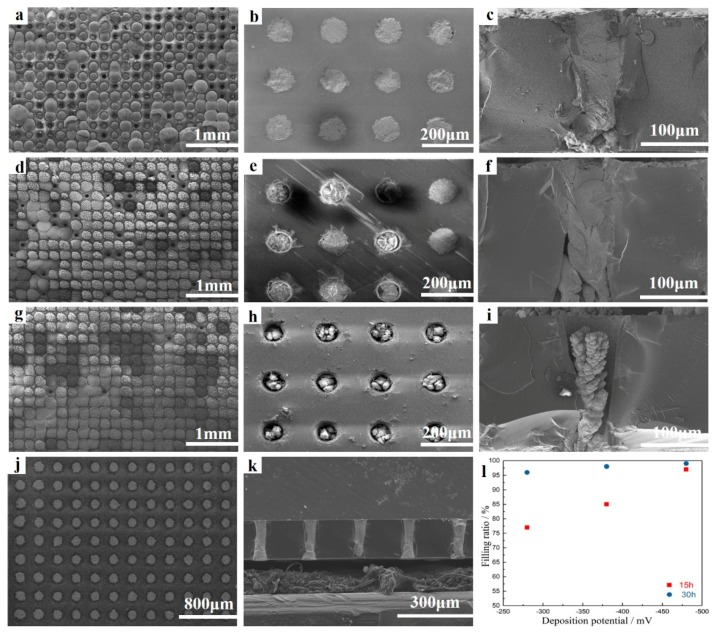
Top-and side-view SEM images of Sb-Te pillars deposited in a glass template at the deposition parameters of (**a**–**c**) −280 mV, 15 h; (**d**–**f**) −380 mV, 15 h; (**g**–**i**) −480 mV, 15 h; and (**j**,**k**) −280 mV, 30 h. (**l**) The filling ratio in micro-pores at the condition of different deposition potential and time.

**Figure 9:**
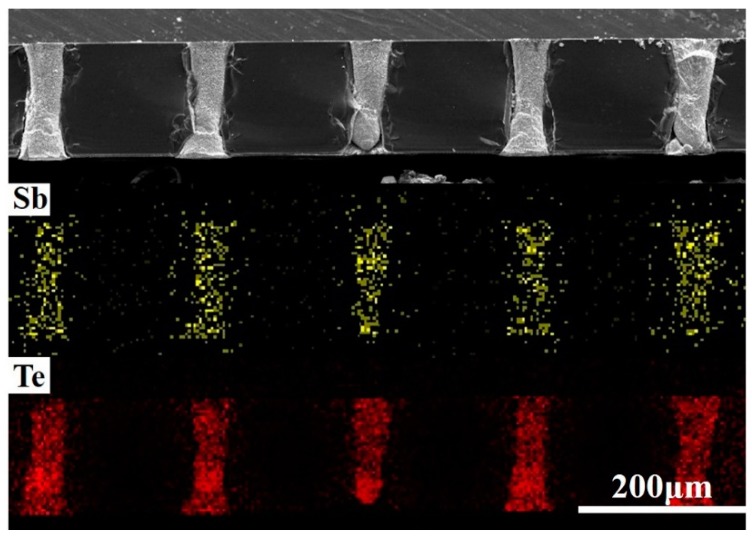
The elementary mappings of the Sb_2_Te_3_ pillars.

Figure 8 depicts SEM images of the micro-pillars deposited in an electrolyte containing 8 mM of SbO^+^ and 12 mM of HTeO^2+^ through pulsed voltage deposition at the reduction potential range from −280 to −480 mV. To observe the inside of micro-pores, the filled template was packaged in epoxy resin and then polished in a metallographic grinding/polishing machine to expose the inside of micro-pores. Figure 8a–i were the top-and side-view SEM images of Sb-Te pillars deposited at the deposition potentials of −280 mV, −380 mV, and −480 mV with 15 h, respectively. It revealed that as the cathode potential moved from −280 mV to −480 mV, the density of deposits decreased, but the speed of nucleation and growth rose and the filling rate increased. This is because the current density and nucleation rate and growth rose due to the higher over-potential and numerous crystal nuclei stuck to the Si wafer-based Pt electrode at the bottom of the template, allowing more micro-pores to be filled. However, the filling quality was decreased because of the high growth rate, which decreased density and led to more filling defects. The concentration gradient became more obvious with increasing over-potential, which caused dendrite growth in crystal grains and the decrease of pillar density. Considering these findings, the cathode reduction potential of −280 mV was selected to obtain the micro-pillars with higher density. Using the three-step pulsed voltage regime illustrated in Figure 6, relatively compact pillar deposits were obtained. In 20 pulsed cycles, the integral charge is −0.018 C from the current-time curve shown in Figure 7c. It takes about 15 h in theory to complete the deposition of an entire micro-pillar. However, actually the growth of each pillar is effected by surrounding pillars; besides, the growth speed of individual micro-pillars is much higher than others on account of a local current density concentration which was raised by nonuniformity in micro-pores forming. To increase the filling ratio, we lengthened the deposition time to ensure that most micro-pores were filled with the TE material. When the deposition time was extended to 30 h, the filling ratio of the template deposited at −280 mV can reached 96%. The SEM images of micro-pillars deposited at −280 mV at 30 h were shown in Figure 8j–k. The filling ratio of micro-pillars in the condition of different deposition potentials and times is shown in Figure 8l. After deposition, abrasive paper and a metallographic grinding/polishing machine were used to remove the excess deposited material, ensuring that the micro-pillars were as long as the micro-pores and the upper surface was smooth. The EDS results showed that the micro-pillar composition was similar to that of the films deposited at the same potential. Figure 9 was the elementary mappings of the Sb_2_Te_3_ pillars deposited at −280 mV at 30 h; the result indicated that the composition of the whole micro-pillars was homogeneous mainly, but at the bottom of the pillars, the composition of Sb/ Te was slightly lower than 2:3, because SbO^+^ has inferior diffusibility compared to HTeO_2_^+^.

**Figure 10:**
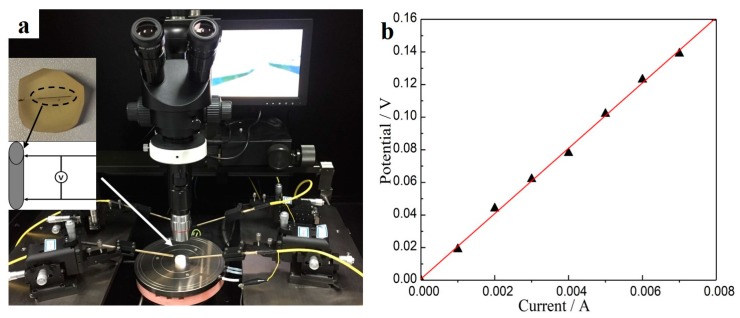
(**a**) The test platform of a single micro-pillar and (**b**) the potential–current curve of a single micro-pillar.

A microprobe test platform was used to measure the potential–current curve of a single pillar to calculate its resistance, as shown in Figure 10. The resistance of the pillar with a length of 180 µm and diameter of 60 µm was about 20 Ω. Considering the Sb_2_Te_3_ films, which have an electrical resistivity of about 10^−6^ Ωm, the same size with the above pillar will have a resistance of approximately 6 Ω. Thus, it seems that a difference exists between the resistances of the micro-pillars and bulk material. The resistance of the micro-pillar was measured by the two-point method using a microprobe, so large contact resistance between the microprobe and micro-pillar is possible. Considering this factor, the measured resistance of the micro-pillar is reasonable. In addition, the crystallinity, grain size, and testing method also influence the resistance of micro-pillars, which remains unsolved and needs more attention in the future. Regardless, these results indicate that micro-pillars of dense Sb_2_Te_3_ with high filling ratio and large aspect ratio can be obtained by electrodeposition based on multi-channel glass template, which can be further applied in the fabrication of micro-TEGs with large output voltage and power.

It seems that the aspect ratio of our deposits is incomparable with the nanowire that has been fabricated by previous work [40,53], but it also takes numerous of nanowire arrays to form a thermoelectric leg when it is assembled into a device. Therefore, the aspect ratio of a single nanowire is meaningless when using TE device. Besides, the lengths of most nanowires are too short to establish a large temperature difference. Therefore, the main advantage of our thermoelectric leg is that it can achieve high integration level in the precondition, such that the length of the TE legs is large enough to establish a large temperature difference.

## 4. Conclusions

Sb_2_Te_3_ TE films and micro-pillars were deposited on Si wafer-based Pt electrode and a microporous glass template, respectively. The results indicate that the composition of deposits depends on the electrolyte concentration, and the micromorphology depends on the reduction potential. With increasing Te concentration, the charge integral and current density of the deposition and dissolution processes both rose. As the deposition potential shifted to negative value, the composition of the deposit remained unchanged, but the grain size increased, and the film density decreased. As a result, Sb_2_Te_3_ film with nominal composition and dense morphology on Si wafer based Pt electrode can be obtained using the reduction potential of −300 mV with 8 mM of SbO^+^ and 12 mM of HTeO_2_^+^. The Seebeck coefficient of aforesaid TE film is positive, which proves that the deposit is a p-type semiconductor. However, it is difficult to obtain compact pillars on the glass template using the same condition on the Si wafer -based Pt electrode because of the concentration polarization when fabricating the micro-pillars. Nevertheless, the pulsed voltage deposition is found to be an effective way to fabricate Sb_2_Te_3_ micro-pillars. A deposition potential of −280 mV and a dissolve potential of 500 mV were selected, which can realize the deposition of micro-pillars with a large aspect ratio and high density. The deposition technology can be further applied in the fabrication of micro-TEGs with large output voltage and power.
